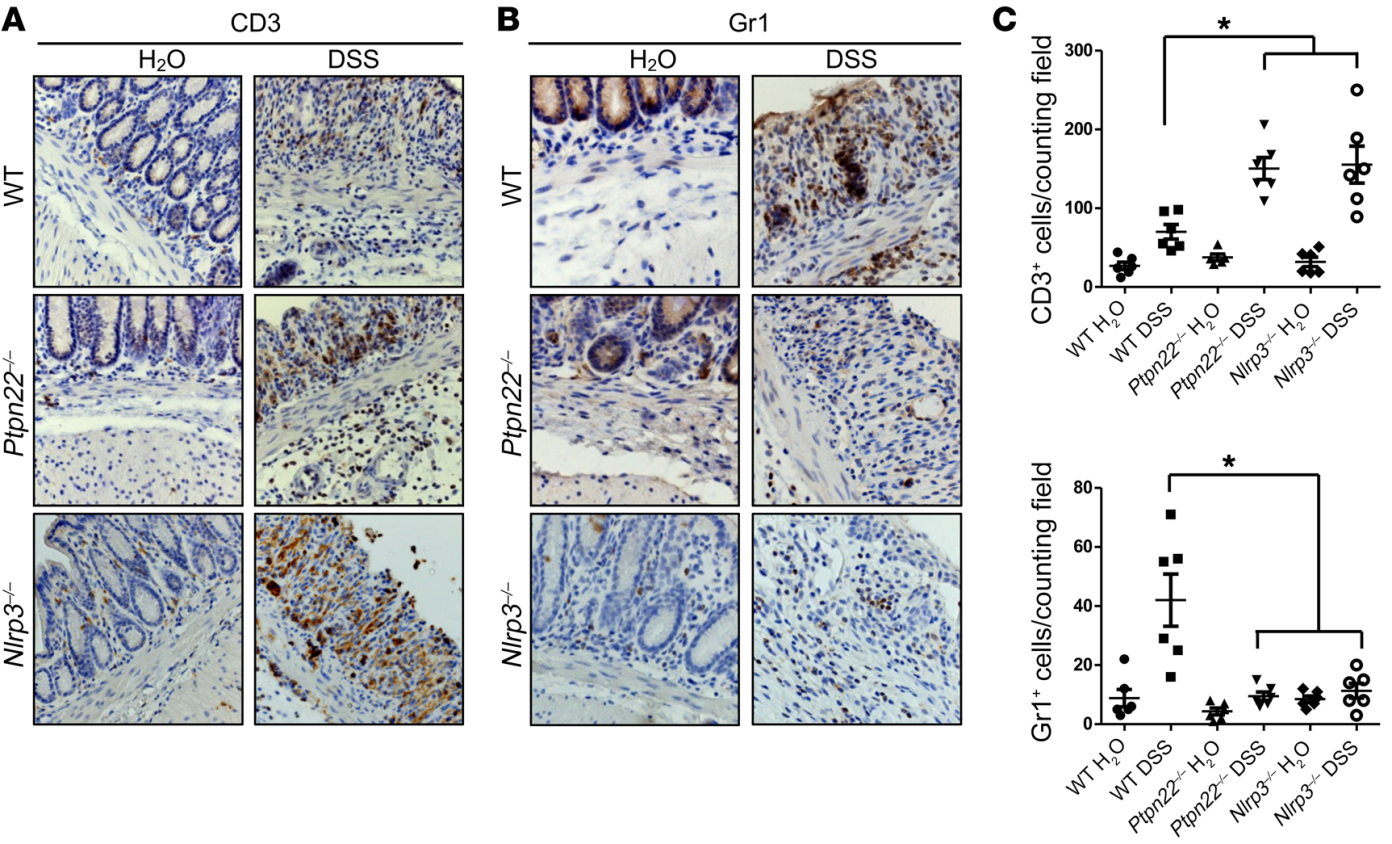# NLRP3 tyrosine phosphorylation is controlled by protein tyrosine phosphatase PTPN22

**DOI:** 10.1172/JCI169304

**Published:** 2023-02-15

**Authors:** Marianne R. Spalinger, Stephanie Kasper, Claudia Gottier, Silvia Lang, Kirstin Atrott, Stephan R. Vavricka, Sylvie Scharl, Petrus M. Gutte, Markus G. Grütter, Hans-Dietmar Beer, Emmanuel Contassot, Andrew C. Chan, Xuezhi Dai, David J. Rawlings, Florian Mair, Burkhard Becher, Werner Falk, Michael Fried, Gerhard Rogler, Michael Scharl

Original citation: *J Clin Invest*. 2016;126(5):1783–1800. https://doi.org/10.1172/JCI83669

Citation for this corrigendum: *J Clin Invest*. 2023;133(4):e169304. https://doi.org/10.1172/JCI169304

The authors recently became aware of errors in Figure 8, A and B. In Figure 8A, the WT/H_2_O and WT/DSS pictures were inadvertently switched. In Figure 8B, the incorrect image was shown for the *Ptpn22^–/–^*/DSS sample. The correct figure is shown below.

The authors regret the errors.

## Figures and Tables

**Figure:**